# A successful pregnancy during the treatment of cervical sarcoma botryoides and advantage of fertility sparing management: A case report

**Published:** 2015-02

**Authors:** Selçuk Ayas, Lutfiye Uygur, Evrim Bostanci, Ayşe Gürbüz

**Affiliations:** 1 *Department of Obstetrics and Gynecology, Zeynep Kamil Women and Children Diseases Research and Training Hospital, Turkey.*; 2 *Department of Gynecology, Kadıköy Florence Nightingale Hospital, Turkey.*

**Keywords:** *Rhabdomyosarcoma*, *Embryonal*, *Cervix*, *Pregnancy*, *Fertility sparing*

## Abstract

**Background:**

Sarcoma botryoides of cervix is a rare variant of rhabdomyosarcomas (RMS) of female genital tract. It is usually diagnosed in first or second decade of life. In this case report, we aimed to present a 21 year-old nulligravid patient who was diagnosed with embryonal RMS of the cervix, to discuss the treatment options that have been stated in the literature, and to highlight the advantage of fertility sparing management in these young patients.

**Case:**

We report a 21-year-old nulligravid woman complaining about a mass protruding from introitus, which was represented with a 8×7 cm “grape-like” cervical polyp on speculum examination. The histopathologic examination of the biopsy taken was combined with immunohistochemical staining with desmin, myogenin, S100, vimentin, and myoglobin. Colposcopy, second biopsy, and positron emission tomography were used during the follow-up. The histopathologic examination revealed embryonal RMS of cervix. She received three cycles of combination chemotherapy, doxorubicin and ifosfamide. She refused to have a surgery because of an unplanned, desired pregnancy at two months after the chemotherapy. She was lost during the follow-up. After having an uneventful pregnancy and a successful delivery, she reapplied at postpartum 6^th^ month. Colposcopic evaluation revealed a local polypoid area, the histopathologic examination of biopsy suggested recurrence even though positron emission tomography scans were unremarkable. Therefore complementary treatment was planned as conization and pelvic lymphadenectomy. The histopathology revealed no residual tumor on the conization material and no involvement of pelvic lymph nodes.

**Conclusion:**

Fertility sparing management, including doxorubicin and ifosfamid combination in chemotherapy step, can be management option. Pregnancy and successful delivery is possible during the treatment. Colposcopy has importance for early detection of recurrences.

## Introduction

Rhabdomyosarcoma (RMS) is an embryonal mesenchyma originated tumor. Although it is the most common soft tissue tumor in childhood and young adulthood, which accounts for 4-6% of all malignancies in these ages, only twenty five percent of these tumors occur in genitourinary tract, and they are mostly reported as vaginal tumors (1-   3). Sarcoma botryoides of cervix is an extremely rare variant of RMS of female genital tract, which has a better prognosis comparing with RMS of uterine corpus and vagina. It is usually diagnosed in first or second decade of life presenting with cervical polyp or rarely infiltrative mass. The Intergroup RMS Study (IRS) defined three major histological subtypes of RMS: alveolar, embryonal, undifferentiated (4). 

Embryonal subtype comprises 50% of the RMS and most commonly originates from the vagina. While vaginal RMS is seen in the first decade of life-generally before two years old, cervical embryonal RMS (sarcoma botryoides) is seen in the second and third decade of life (ranges in age between 3 and 32, mean and median age at diagnosis is 13) usually presenting with a submucosal grape like polypoid lesion or rarely infiltrative mass in cervix (5-7). We report a 21-year-old woman who has cervical RMS, her successful pregnancy and delivery during the treatment and fertility sparing management we performed.

## Case report

A 21-year-old nulligravid patient who had complaints about the feeling of a mass protruding from the vaginal introitus was referred to our clinic. She had had a polypectomy the histopathologic evaluation of which had suggested fibro epithelial polyp and had been recommended no further treatment one year ago. Nine months after her first admittance, she complained about irregular bleeding. On the speculum examination, a macroscopically grape-like polypoid mass in 8×7 cm diameters, which was arising from cervix and filling the lateral fornix was detected and biopsy from this mass was taken. An informed consent has been taken from the patient to report this case.

The histopathologic examination revealed the features of a zone of increased cellularity composed of undifferentiated rhabdomyoblasts beneath the surface epithelium on a background of edematous stroma and cambium layer. Immunohistochemical stainings with desmin, myogenin, S100, vimentin, aand myoglobulin, were positive on a rhabdomyoblastic differentiated cells. CD34 and actin stainings were positive on vessel walls. The histopathologic diagnosis was reported as embryonal RMS relying on these findings. MR imaging and CT scan revealed a heterogeneous cervical mass 7×6×6 cm in diameter whose margins cannot clearly differentiated from vagina, but no metastatic disease. She was recommended to receive three cycles of neoadjuvant chemotherapy and subsequent conization with laparoscopic lymph node dissection in order to preserve fertility. 

She was administered three courses of chemotherapy, with doxorubicin 150 mg daily, ifosfamid 4 gr daily and mesna 3600 mg daily. She received three courses with three weeks intervals without any delay. Four weeks after neoadjuvant therapy, she was admitted for the operation as planned, however her thyroid functions were impaired and inadequate for surgery. Hence, the surgery was delayed three weeks to adjust the thyroid functions medically. During this period, she became pregnant incidentally so did not accept any further treatment. She had an uneventful pregnancy and a successful caesarean section on term. She did not apply for follow up until sixth month after delivery. When she reapplied, colposcopic evaluation revealed a local polypoid area less than 1 cm in diameter. The histopathologic examination of biopsy from this area suggested recurrence even though positron emission tomograph scan was unremarkable. Therefore complementary treatment was performed as simple conization with laparoscopic pelvic lymph node dissection. The final histopathology results revealed no residual tumor on the conization material and no involvement of pelvic lymph nodes. Follow up is planned as colposcopic examination every six months in the first year. 

**Figure 1:**
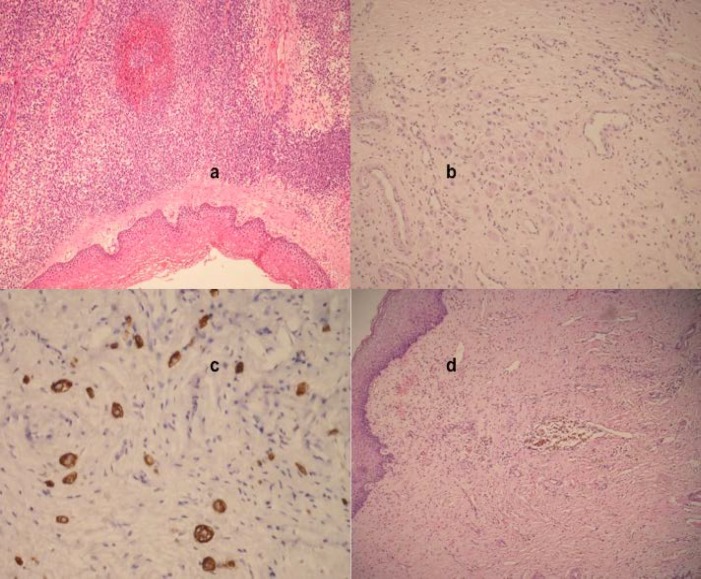
a) First vaginal biopsy, embryonel RMS (HEX100); b) Rhabdomyoblastic cells in cervical biopsy after chemotherapy (HEX100); c) Strong desmin positivity in residual cells (Desminx200); d) No residual material in conization material (HEX100)

## Discussion

Since 1950s, treatment of sarcoma botryoides has dramatically changed. In the late 1950s and 1960s pelvic exenteration was recommended to be the first choice of therapeutic approach whereas today, treatment modalities range from polypectomy, conization, cervicectomy, trachelectomy up to radical hysterectomy depending on the extension of the tumor and necessity for fertility preserving (8). Brand *et al* put forward that cervical sarcoma botryoides has a similar prognosis to the other embryonal RMS of the genital tract and that chemotherapy and response-dependent surgery should be the treatment of choice in 1987. However, in 1988, Daya and Scully reported that sarcoma botryoides of cervix has a more favorable outcome comparing with its counterpart in vagina or uterus and polypoid lesions have better prognosis, hence local excision procedures followed by chemotherapy may be considerable for these patients (9). 

In 1990s, treatment modalities have mostly been shifted towards organ sparing surgeries like local excision, polypectomy, cervicectomy, conization with or without chemotherapy so that they could abandon radical surgery associated morbidity. The survival rates increased from 25% in 1975 to 65% in 1995 (10). This fertility preserving approach was also supported by the review study by Zeisler *et al* (11). After these encouraging studies many cases managed by organ preserving approach have been reported (12-17). There are chemotherapy regimens, which are used in adjuvant or neoadjuvant settings in order to improve the outcome of the treatment. Although vincristine, actinomycin and cyclophosphamide containing regimes are the mostly preferred chemotherapy, we chose the combination of doxorubicin and ifosfamide in a neoadjuvant setting because of its shown effectiveness in conservative treatment by Zanetta *et al* (18). 

Neoadjuvant setting was also for reducing the tumor size to be able to apply a fertility preserving surgery. It was also reported that normal menstrual function start again between 36 and 38 months following initial diagnosis in three cases treated with this combination (18). In our cases normal menstrual function was started again in two weeks after chemotherapy treatment. Uneventful period of pregnancy and delivery with a normal baby occurred. Our case presents a good example that contraception should be considered during and in the early period of the chemotherapy treatment and the possibility of normal pregnancy outcome by using the regime. All these possibilities should be included in the information given to the patients. The importance of the clinical follow-up to detect possible failure of the treatment and unfavorable prognosis has been stated in the studies (19, 20). 

In our case cervical smear was negative but colposcopy directed biopsies were positive. This was also in favor of the conclusion of the study which suggest the use of updated follow-up techniques for early detection of persistent/recurrent tumor by Zanetta *et al* (18). No tumor of the cervix was associated with the involvement of regional lymph nodes have not been reported in the study which evaluated Intergroup RMS Study Group (IRSG) protocols I-IV (21). We decided to evaluate lymph node situation by means of a minimal invasive method, laparoscopic pelvic lymphadenectomy due to the time gap between primary chemotherapy and surgical intervention and the radiotherapy need in case of presence of positive lymph node.

## Conclusion

In conclusion the median age of the patients with sarcoma botryoides obligates us to keep closer to fertility sparing managements. Fertility preserving can be a possible option in these cases. Doxorubicin and ifosfamid combination can be an option in chemotherapy step of fertility sparing management. Close follow-ups with colposcopy have utmost importance for early detection of recurrences. 

## Conflict of interest

We declare that we have no conflict of interest. 
